# Large Language Model‐Driven Analysis and Report Generation of Endoscopy Videos—A Pilot Study

**DOI:** 10.1111/den.70134

**Published:** 2026-03-10

**Authors:** Davide Massimi, Luca Di Stefano, Tommy Rizkala, Marco Spadaccini, Yuichi Mori, Maddalena Menini, Giulio Antonelli, Kareem Khalaf, Raf Bisschops, Daniel von Renteln, Prateek Sharma, Douglas K. Rex, Michael Bretthauer, Carlo Castoro, LLM Working Group, Roberto De Sire, Ludovico Alfarone, Alessandro D’Aprano, Silvia Carrara, Roberta Maselli, Vincenzo Vadalà, Francesco Menini, Abdelrahman Ashraf Alawdy Elsaman, Alessandro Fugazza, Matteo Colombo, Renato de Martino, Antonio Capogreco, Gianluca Franchelucci, Victor Savevski, Elena De Momi, Luca Carlini, Chiara Lena, Sravanthi Parasa, Susanne O’Reilly, Simone Dibitetto, Matteo Spertino, Alessandro Repici, Cesare Hassan

**Affiliations:** ^1^ IRCCS Humanitas Research Hospital Milan Italy; ^2^ Clinical Effectiveness Research Group University of Oslo Oslo Norway; ^3^ Digestive Disease Center Showa University Northern Yokohama Hospital Yokohama Japan; ^4^ Gastroenterology and Digestive Endoscopy Unit Ospedale dei Castelli Rome Italy; ^5^ Division of Gastroenterology, St. Michael's Hospital University of Toronto Toronto Ontario Canada; ^6^ Department of Gastroenterology and Hepatology University Hospitals Leuven, TARGID, KU Leuven Leuven Belgium; ^7^ Montreal University Hospital Research Center Montreal Quebec Canada; ^8^ Division of Gastroenterology Montreal University Hospital Center (CHUM) Montreal Quebec Canada; ^9^ University of Kansas School of Medicine and VA Medical Center Kansas City Missouri USA; ^10^ Division of Gastroenterology Indiana University School of Medicine Indianapolis Indiana USA; ^11^ Department of Biomedical Sciences Humanitas University Milan Italy

**Keywords:** artificial intelligence, digestive system, endoscopy, gastrointestinal, natural language processing

## Abstract

Multimodal large language models (MLLMs) can automatically analyze clinical video, but evidence from full esophagogastroduodenoscopy (EGD) and the impact of on‐screen computer‐aided detection/diagnosis (CAD) overlays on MLLM behavior remain unclear. We tested whether an MLLM can produce clinically adequate EGD reports and whether a CAD overlay changes performance. We analyzed five complete EGD videos with Gemini 2.5 Pro in paired versions: (1) clean video and (2) the same video with a CAD overlay. Five blinded endoscopists rated report adequacy in three domains. MLLM accuracy for landmarks/lesions was further assessed by two blinded expert endoscopists using the time‐window rule (a model detection counted as correct if it occurred within ±2 s of the expert‐annotated timestamp). In this retrospective pilot study, five archived diagnostic EGD procedures from five patients were available as full‐length videos. Across five raters, MLLM Completeness was judged adequate in 56.0% (14/25 ratings) with Clean‐Video versus 48.0% (12/25 ratings) with Overlay‐Video (*p* = 0.500). Visualization was identical (36.0% [9/25 ratings] for both; *p* = 1.000). Lesions characteristics were identical (16.0% [4/25] for both; *p* = 1.00). For the Landmark agreement, the overall accuracy of the MLLM with Clean‐Video vs. Overlay‐Video was: 0.55 [95% CI 0.43–0.67] vs. 0.33 [0.23–0.46], *p* = 0.029; sensitivity 0.53 [0.40–0.66] vs. 0.35 [0.24–0.49], *p* = 0.122; specificity 0.67 [0.35–0.88] vs. 0.22 [0.06–0.55], *p* = 0.125. In this pilot study, Gemini 2.5 Pro demonstrated inadequate performance for clinical EGD reporting. These hypothesis‐generating findings suggest substantial optimization and larger‐scale validation are required before deployment.

## 1 Introduction

Standardized, complete reporting is essential to high‐quality esophagogastroduodenoscopy (EGD), yet documentation quality and lesion reporting remain variable in practice [1, 2]. Multimodal large language models (MLLMs) can process long clinical videos and generate structured and narrative outputs, raising the possibility of near–real‐time automated endoscopy reports [3]. In colonoscopy, early studies using public datasets have shown that M‐LLMs can approach good performance for polyp morphology classification and polyp detection, though limitations remain [4, 5]. However, most prior evaluations have focused on still images or short clips rather than full procedures, and it is unclear whether on‐screen computer‐aided detection/diagnosis (CAD) overlays influence MLLM behavior when interpreting endoscopy video. We therefore conducted a paired, same‐video study to answer two clinically relevant questions: (1) can an MLLM produce a clinically adequate report for a complete EGD, and (2) does adding a CAD overlay to the identical video stream change landmark recognition, lesion identification, or report adequacy?

## 2 Procedure

### 2.1 Study Design

For this retrospective pilot analysis, we identified five adult patients who had undergone diagnostic EGD at our institution and for whom full‐length procedure videos were available in the institutional archive.

We performed a within‐case paired analysis of the five complete EGD procedures. For each case, two synchronized inputs were analyzed: the clean video (Clean‐Video) and the same video with an EndoAngel CAD overlay (Overlay‐Video) that presented additional real‐time information on the endoscopy screen. Prompts and analysis settings were prespecified and kept identical in the two types of videos.

### 2.2 Data Extraction and Model Prompting

Video acquisition and preprocessing are reported in Appendix S1.

The eligibility criteria for video selection were: (i) complete recording from intubation to withdrawal; (ii) standard diagnostic adult EGD (no major therapeutic interventions or complications); and (iii) availability of both the clean video and the version with the EndoAngel CAD overlay. Videos with incomplete recording, major artifacts, or prior upper‐GI surgery were excluded. Video recording is a routine practice at our institution for quality assurance. All videos were fully anonymized before cloud upload; Gemini's policies specify user content is not used for model training.

**FIGURE 1:**
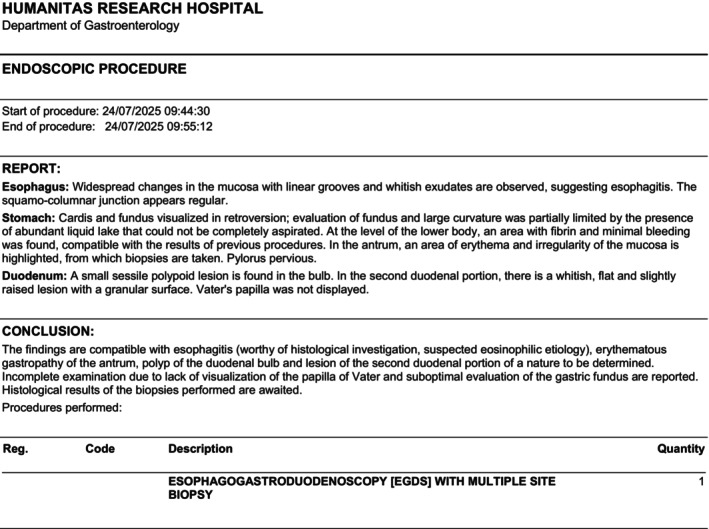
Example of a generated report by Gemini 2.5 Pro.

Identical prompts/settings were used for Clean‐Video and Overlay‐Video, prompt details are available in Appendix S2. The two videos were processed with Gemini 2.5 Pro (Google AI Studio; temperature 0.0, top‐*p* 0.95, max tokens 65,536) using a two‐step workflow: (1) structured extraction (JSON) with predefined fields capturing the 12 ESGE upper‐GI landmarks (first occurrence and focused inspection), mucosal visibility, procedural completeness, and discrete lesion descriptors [1]; (2) narrative reporting, in which the model generated a case‐level report by explicitly referencing its own JSON and imitating the style/structure of real, anonymized reports (Figure 1).

### 2.3 Reference Standard and Rating

Five endoscopists first watched the video for each case and, with the input condition masked (Clean‐Video vs. Overlay‐Video), rated two model‐generated reports per case for each video as Adequate/Inadequate across three domains:

- *Completeness*: indication; extent/landmark coverage; findings with location and size descriptors; interventions; complications/adverse events; conclusion/recommendations. Missing any major element implies an inadequate evaluation.
- *Visualization*: global judgment of mucosal cleanliness/visibility and adequacy of documentation for all relevant areas (e.g., adequate insufflation and views including retroflexion when appropriate). Insufficient visualization or missing critical views implies an inadequate evaluation.
- *Lesions characteristics* (reporting domain): correct identification and description of any discrete lesions with site, morphology, and size/estimate as appropriate. Omission or incorrect attribution leads to an inadequate evaluation. Experts identified all mucosal abnormalities warranting clinical documentation (erosions, ulcers, polyps, masses, and suspicious areas); minor findings not altering management were excluded.

In parallel, two senior endoscopists independently reviewed all videos and completed the same structured schema; in other words, they answered the same questions that the MLLM had to answer. Disagreements between the two experts were resolved under a liberal consensus rule: a landmark/event was considered present if at least one expert marked it present, recognizing that the occurrence could have been annotated on insertion (intubation) or on withdrawal; otherwise, it was considered absent.

### 2.4 Landmark Agreement

We evaluated landmark agreement using a temporal‐overlap criterion to reflect clinical conditions, because the timing of landmark identification naturally varies with endoscopic maneuvers, insufflation, and viewing angle. We selected ±2 s based on typical focused inspection duration (1–3 s) and inherent inter‐annotator variability. For each expert‐annotated landmark, an MLLM detection was considered correct if the temporal window around the predicted timestamp ([predicted_timestamp ±2 s]) overlapped with the temporal window around the expert timestamp ([expert_timestamp ±2 s]), implementing a bidirectional range‐overlap criterion. Operational definitions: true positive (TP) = detection within the overlapping temporal windows; false positive (FP) = detection outside the window or for landmarks absent by expert reference; false negative (FN) = missed expert‐annotated landmark; true negative (TN) = landmark absent in expert consensus and not detected by the MLLM.

### 2.5 Outcomes

Each case contributed two reports—one derived from Clean‐Video and one from Overlay‐Video—rated by five blinded endoscopists.

The primary outcome was adequacy (Adequate/Inadequate) of the MLLM‐generated case report across the predefined domains: Completeness, Visualization, and Lesions.

Secondary outcomes were: (a) Landmark agreement with the expert reference using a prespecified temporal‐overlap rule: a model detection counted as correct when it occurred within ±2 s of the expert‐annotated timestamp for that landmark. Agreement was summarized at the level of all 12 ESGE upper‐GI landmarks across all cases and, separately, by organ (esophagus, stomach, duodenum). (b) Per‐video lesion detection was calculated using expert‐confirmed lesions as denominators.

### 2.6 Statistical Analysis

#### 2.6.1 Primary Outcome

Paired differences in adequacy (Clean‐Video vs. Overlay‐Video) used exact McNemar tests. Inter‐rater reliability was quantified with Fleiss' κ per domain. A logistic mixed‐effects model (GLMM) estimated overlay effects with condition (Clean‐Video vs. Overlay‐Video) as fixed effect and random intercepts for rater and video; effects are reported as odds ratios (ORs) with 95% CIs. Binomial 95% confidence intervals for adequacy rates were computed using the exact Clopper–Pearson method.

#### 2.6.2 Secondary Outcomes

For the landmark agreement, we computed accuracy, sensitivity, and specificity across all 12 × 5 = 60 video×landmark pairs with Wilson 95% confidence intervals (more accurate for proportions than normal approximation). Paired comparisons between video 1 and video 2 were performed using McNemar's test, a non‐parametric test for paired binary data that focuses on discordant pairs. McNemar's test was applied separately for each metric: [1] accuracy (overall correct classifications: tp + tn vs. fp + fn), [2] sensitivity (correct detections among cases with ground truth: tp vs. fn), and [3] specificity (correct rejections among cases without ground truth: tn vs. fp). For organ‐level subgroup analyses with small sample sizes (*n* ≤ 30), Fisher's exact test was used instead of McNemar's test when appropriate due to sparse contingency tables.

For lesion detection rates, exact binomial confidence intervals were calculated using the Clopper–Pearson method. Paired comparisons used binomial exact tests due to small sample sizes (*n* = 2–3 lesions per video).

#### 2.6.3 Sensitivity Analysis

To validate robustness, we performed a sensitivity sweep varying the tolerance window from 1 to 5 s on either side of the expert timestamp.

#### 2.6.4 Statistical Software

All analyses were performed using Python 3.x with scipy.stats for hypothesis testing. A two‐sided significance level of α = 0.05 was used throughout. No correction for multiple comparisons was applied, as primary and secondary outcomes were prespecified, and organ‐level analyses were considered exploratory.

## 3 Results

### 3.1 Cohort and Video Characteristics

Five patients undergoing diagnostic EGD contributed to five full‐length videos (one per patient); mean age was 64 years (range 29–80), and 4/5 were male. Indications for EGD included dyspepsia (*n* = 2), Barrett's esophagus surveillance (*n* = 1), anemia workup (*n* = 1), and epigastric pain (*n* = 1). All patients tested negative for *Helicobacter pylori* by rapid urease test. All procedures were performed under propofol sedation. Procedure duration averaged 7 min and 58 s (range 4:39–10:50). No periprocedural complications were recorded. Across all videos, experts identified a total of 14 discrete lesions (by case: VID01 *n* = 2; VID02 *n* = 3; VID03 *n* = 3; VID04 *n* = 3; VID05 *n* = 3). These counts define the denominators used for per‐video lesion detection rate. All 10 video inputs (5 Clean‐Videos and 5 Overlay‐Videos) were successfully processed by the MLLM without system freezes or aborted runs, yielding a technical success rate of 100% for long‐video analysis in this pilot. Median processing time per video on the cloud infrastructure was approximately 1.3 min (range 1.0–2.3), and no hardware‐ or memory‐related failures were observed.

### 3.2 MLLM Performance on EGD Video Analysis

#### 3.2.1 Report of Adequacy by Domain (See Table 1)

**TABLE 1:** Main results: primary outcome and secondary outcomes.

Primary outcome					
	Subdomain	Clean‐Video	Overlay‐Video	*p* *	Fleiss' κ (interp.)
Report adequacy by domain (5 raters)	Completeness [95% CI]	14/25, 56.0% [34.9–75.6]	12/25, 48.0% [27.8–68.7]	0.500	0.079 (Slight)
	Visualization [95% CI]	9/25, 36.0% [18.0–57.5]	9/25, 36.0% [18.0–57.5]	1.000	0.002 (Slight)
	Lesions characteristics [95% CI]	4/25, 16.0% [4.5–36.1]	4/25, 16.0% [4.5–36.1]	1.000	0.107 (Slight)

Secondary outcomes					
	Metric	Clean‐Video	Overlay‐Video	*p* *	Sample^a^
Landmark agreement (Δ = 2 s tolerance)	Accuracy [95% CI]	55% [43–67]	33% [23–46]	0.029****	60
	Sensitivity [95% CI]	53% [40–66]	35% [24–49]	0.122	60
	Specificity [95% CI]	67% [35–88]	22% [6–55]	0.125	60
Per‐Video lesion detection	**Video case** ^a^	**Clean‐Video**	**Overlay‐Video**	***p*** **	**Δ (Video 2 – Video 1), %**
	VID01	0/2, 0.0% [0.0–84.2]	0/2, 0.0% [0.0–84.2]	1.000	0.0
	VID02	2/3, 66.7% [9.4–99.2]	2/3, 66.7% [9.4–99.2]	1.000	0.0
	VID03	0/3, 0.0% [0.0–70.8]	3/3, 100.0% [29.2–100.0]	0.250	+100.0
	VID04	0/3, 0.0% [0.0–70.8]	0/3, 0.0% [0.0–70.8]	1.000	0.0
	VID05	1/3, 33.3% [0.8–90.6]	2/3, 66.7% [9.4–99.2]	0.259	+33.3
	Total	3/14, 21.4% [4.7–50.8]	7/14, 50.0% [23.0–77.0]	1.000***	+28.6

*Note:* Landmark agreement (Δ = 2 s tolerance): accuracy shows significant deterioration with cad overlay (*p* = 0.029), while sensitivity and specificity show non‐significant trends toward deterioration (*p* = 0.122 and *p* = 0.125, respectively), likely due to high variability across organs and limited true negative cases (*n* = 9). Video 1 = Clean video; Video 2 = With CAD overlay.

* *p*‐values for paired comparisons using McNemar's test.

** *p*‐values calculated using binomial test for paired comparison. Confidence intervals are exact binomial (Clopper‐Pearson method).

*** Total *p*‐value reflects aggregate comparison; individual *p*‐values may not be directly comparable due to small sample sizes.

**** Statistical significance (*p* < 0.05).

^a^ Sample size: 60 landmark × video pairs (12 landmarks × 5 videos).

Across five raters, MLLM Completeness was judged adequate in 56.0% (14/25) with Clean‐Video versus 48.0% (12/25) with Overlay‐Video; the within‐pair difference was not significant (McNemar *p* = 0.500). Inter‐rater agreement was slight (Fleiss' κ = 0.079).

For Visualization, MLLM adequacy was identical with both videos (36.0% [9/25] for both Clean‐Video and Overlay‐Video; McNemar *p* = 1.000), with slight agreement (Fleiss' κ = 0.002).

For Lesions, MLLM adequacy was also identical with Clean‐Video and Overlay‐Video (16.0% [4/25]; McNemar *p* = 1.000), with slight agreement (Fleiss' κ = 0.107).

The generalized linear mixed model (GLMM) analysis confirms the findings from McNemar's test: no significant effect of CAD overlay on report adequacy (OR = 0.80, 95% CI 0.11–5.92, *p* = 0.825); GLMM results for report adequacy are presented in Appendix S3.

#### 3.2.2 Landmark Agreement

**FIGURE 2:**
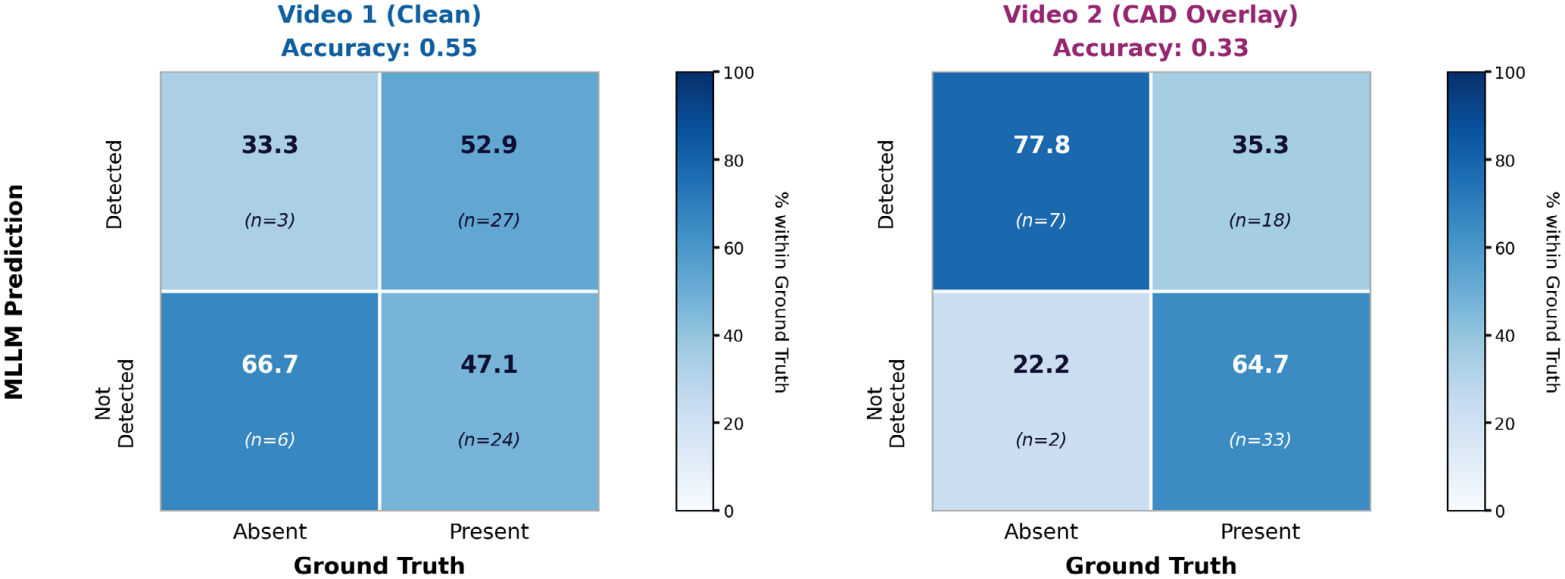
Normalized confusion matrices and summary metrics at Δ = 2 s comparing Clean‐Video vs. Overlay‐Video.

Using the temporal‐overlap criterion at the two‐second tolerance window and evaluating 12 × 5 = 60 video × landmark pairs, the overall accuracy of MLLM was higher with Clean‐Video vs. Overlay‐Video (0.55 [95% CI, 0.43–0.67] vs. 0.33 [0.23–0.46]; McNemar *p* = 0.029). Sensitivity was 0.53 (0.40–0.66) for Clean‐Video vs. 0.35 (0.24–0.49) for Overlay‐Video (*p* = 0.122), and specificity was 0.67 (0.35–0.88) vs. 0.22 (0.06–0.55) (*p* = 0.125), respectively (see Table 1 and Figure 2). The organ‐level landmark agreement varied across the esophagus, stomach, and duodenum (see Table S1). In the esophagus, MLLM accuracy was 0.85 with Clean‐Video versus 0.50 with Overlay‐Video (Fisher's *p* = 0.041); sensitivity was 0.85 vs. 0.50 (*p* = 0.041); specificity was not available. In the stomach, MLLM accuracy was 0.30 with Clean‐Video vs. 0.23 with Overlay‐Video (*p* = 0.804); sensitivity 0.25 vs. 0.29 (*p* = 1.000); specificity 0.50 vs. 0.00 (*p* = 0.182). In the duodenum, MLLM accuracy was 0.70 with Clean‐Video vs. 0.30 with Overlay‐Video (*p* = 0.179); sensitivity 0.57 vs. 0.14 (*p* = 0.266); specificity 1.00 vs. 0.67 (*p* = 1.000). Landmark coverage varied by video: not all 12 landmarks were visible in all 5 videos according to expert consensus (see Table S2 for ground‐truth coverage rates per landmark).

#### 3.2.3 Per‐Video Lesion Detection

At the per‐video level, total lesion detection rate was 21.4% (3/14) for Clean‐Video versus 50.0% (7/14) for Overlay‐Video, with an absolute difference of +28.6 percentage points in favor of Overlay‐Video. The individual lesion detection data per video is reported in Table 1.

### 3.3 Sensitivity Analysis (Tolerance‐Window Sweep)

As prespecified, we varied the tolerance window from 1 to 5 s on either side of the expert timestamp; methods and full results are presented in Figure S1 and Table S3.

## 4 Discussion

This study provides a comprehensive and pragmatic evaluation of a state‐of‐the‐art multimodal large language model for automated reporting of upper gastrointestinal endoscopy. Our evaluation focused on landmark recognition, visualization documentation, and lesion detection/characterization. The system was not tested for severity grading (e.g., gastritis classification, Barrett's extent). Future implementation would require task‐specific fine‐tuning and validation against histopathological standards.

Automated case‐level reporting of full‐length EGDs by Gemini 2.5 Pro was technically feasible but clinically underperforming, with only about half of reports judged adequate across the key domains of landmarks, visualization, and lesions. Adding a CAD overlay on the endoscopy screen increased lesion detection but reduced landmark accuracy and specificity, and did not translate into better overall report adequacy. Given this performance, most clinicians would regard these results as poor and clinically unacceptable.

### 4.1 Technical Feasibility Versus Clinical Utility

From a technical standpoint, this work demonstrates the real‐world feasibility of end‐to‐end MLLM‐driven video analysis within standard cloud infrastructure, with minimal computational demands and high model confidence regardless of input type. Such scalability is a key prerequisite for broad clinical translation. However, our findings highlight that technical feasibility alone is insufficient: without high clinical accuracy and reliability, an apparently “working” pipeline remains unsafe and unusable in practice.

### 4.2 Clinical Perspective

From a clinician's perspective, automated drafts are most valuable when they reduce cognitive and documentation load without introducing new reconciliation work. Our results indicate that clinicians would still need to (a) verify landmarks, (b) fill gaps in visualization adequacy, and (c) reconcile lesion characteristics. In this study, the MLLM failed to consistently meet these expectations. Without improvements in these areas, net time savings are limited, and automation bias (accepting machine‐suggested findings without full verification) becomes a safety concern. In addition, the “CAD overlay” video's higher lesion detection did not translate into better adequacy on these dimensions. This nuanced effect underscores that overlays originally designed for human interpretation do not automatically translate into universal AI benefit. In other words, Human‐Machine interaction is different from machine–machine interaction.

### 4.3 Engineering Perspective

From an engineering perspective, the CAD overlay degradation can be explained by: (1) visual information overload—overlay graphics increase visual token density, competing for finite attention capacity; (2) signal‐to‐noise degradation—high‐contrast synthetic elements receive disproportionate attention weights; (3) training distribution mismatch—MLLMs trained on clean images find overlay conditions out‐of‐distribution. Unlike humans, who cognitively filter familiar CAD elements, MLLMs lack this capability. Overlays designed for human interpretation do not automatically benefit machine vision; future systems may require machine‐readable formats or separate processing streams.

### 4.4 Future Optimization

Several optimization strategies could improve the model performance: (1) task‐specific fine‐tuning on annotated endoscopic datasets; (2) structured output constraints aligned with established classifications (ESGE, Paris, Kyoto); (3) overlay‐aware architectures; (4) hybrid human–AI workflows with MLLM‐generated drafts for rapid verification.

### 4.5 Limitations

This study has several limitations. First, the small sample (*n* = 5), while appropriate for pilot feasibility assessment, limits statistical power and generalizability. The findings should be considered hypothesis‐generating. Second, we tested one MLLM (Gemini 2.5 Pro) and one CAD system (EndoAngel); conclusions apply only to this configuration. Third, lesion assessment used expert consensus rather than pathology confirmation. Fourth, our *H. pylori* ‐negative cohort limits generalizability to populations with higher infection prevalence [6]. Finally, our ESGE‐based evaluation may not capture regional practice variations; Japanese guidelines emphasize different documentation patterns (e.g., Kyoto classification).

Nonetheless, several design features strengthen internal validity: analysis of full‐length procedures with prespecified prompts, paired inputs, blinded ratings, and an expert reference for landmarks and lesions. The pre‐planned tolerance sweep (±1–5 s) yielded qualitatively consistent contrasts between conditions, supporting robustness of the main findings.

## 5 Conclusion

This pilot study provides preliminary evidence that the current MLLM configuration does not meet performance thresholds for clinical EGD reporting. Given the small sample size (*n* = 5), findings are hypothesis‐generating. Larger validation studies are needed before clinical implementation.

## Author Contributions

D.M., L.D.S., T.R., C.H.: conception and design; D.M., L.D.S., T.R., Y.M., C.H., M.S.: data extraction and interpretation; L.D.S.: statistical analysis; D.M., L.D.S., T.R.: drafting of the article; All authors: critical revision of the article for important intellectual content. All authors read and approved the final version of the manuscript.

## Funding

Cesare Hassan and Alessandro Repici are supported by the European Commission (Horizon Europe 101057099). Fondazione AIRC per la ricerca sul cancro ETS: IG 2022—ID 27843 project/(AIRC) IG 2023—ID 29220 project and Bando PNRR‐MCNT2‐2023‐12377041. Yuichi Mori is supported by the European Commission (Horizon Europe: 101057099). Michael Bretthauer is supported by the European Commission (Horizon Europe No. 101057099), and Norwegian Research Council (grant 36935, 315410). Raf Bisschops is supported by a grant of research foundation Flanders (G072621N) and KU Leuven.

## Ethics Statement

The study was approved by the Lombardia Ethics Committee CET 5 (Fathom Protocol No. 773/25). Informed consent was waived due to the retrospective nature of the study and use of anonymized data. All videos were anonymized before analysis, and raters were blinded to video conditions. No animal studies were conducted. Clinical trial registration was not applicable for this retrospective analysis.

## Conflicts of Interest

Cesare Hassan: Fujifilm Co. (consultancy); Medtronic Co. (consultancy). Alessandro Repici: Fujifilm Co. (consultancy); Olympus Corp (consultancy); Medtronic Co. (consultancy). Yuichi Mori: Olympus Corp (consultancy, speaking honorarium, equipment loan); Cybernet System Corp. (loyalty). Raf Bisschops: research grants and speaker fees from Medtronic, Fujifilm, and Pentax. Prateek Sharma: consultancy to Boston Scientific and Olympus Inc. and has received grant support from US Endoscopy, Medtronics, Fujifilm, Ironwood, Cosmo Pharmaceuticals, and Erbe.

## Supporting information


**Table S1:** Landmark agreement by organ.
**Table S2:** Ground‐truth coverage by landmark.
**Table S3:** Accuracy metrics across temporal tolerance windows.
**Figure S1:** Temporal sensitivity analysis: Performance vs. Tolerance windows.
**Figure S2:** Representative example of inadequate MLLM output demonstrating common failure modes.
**Figure S3:** Analysis workflow for MLLM‐driven EGD report generation.
