# Supplementary material for: Large Language Model‐Driven Analysis and Report Generation of Endoscopy Videos—A Pilot Study
Source: Dig Endosc. 2026 Mar 10;38(3):e70134. doi: 10.1111/den.70134 (PMC12972633; doi:10.1111/den.70134)
Supplement: Supplementary file 1 — Table S1: Landmark agreement by organ. Table S2: Ground‐truth coverage by landmark. Table S3: Accuracy metrics across temporal tolerance windows. Figure S1: Temporal sensitivity analysis: Performance vs. Tolerance windows. Figure S2: Representative example of inadequate MLLM output demonstrating common failure modes. Figure S3: Analysis workflow for MLLM‐driven EGD report generation. [file DEN-38-0-s001.docx]

# SUPPLEMENTARY MATERIAL

Table of Contents

[SUPPLEMENTARY MATERIAL 1](#_Toc221120044)

[APPENDIX 1: VIDEO ACQUISITION AND PREPROCESSING 1](#_Toc221120045)

[APPENDIX 2: MODEL PROMPTING PROTOCOL 1](#_Toc221120046)

[APPENDIX 3: GLMM results for report adequacy 4](#_Toc221120047)

[Supplementary Tables 5](#_Toc221120048)

[Supplementary Table S1. Landmark agreement by organ 5](#_Toc221120049)

[Supplementary Table S2. Ground-truth coverage by landmark 6](#_Toc221120050)

[Supplementary Table 3. Accuracy metrics across temporal tolerance windows 6](#_Toc221120051)

[SUPPLEMENTARY FIGURES 8](#_Toc221120052)

[Supplementary Figure S1. Temporal sensitivity analysis: Performance vs Tolerance windows: 8](#_Toc221120053)

[Supplementary Figure S2. Representative example of inadequate MLLM output demonstrating common failure modes. 9](#_Toc221120054)

[Supplementary Figure S3. Analysis workflow for MLLM-driven EGD report generation. 10](#_Toc221120055)

# APPENDIX 1: VIDEO ACQUISITION AND PREPROCESSING

Procedures were recorded on standard FUJIFILM endoscopy systems (FUJIFILM Corporation, Tokyo, Japan) at 1080p/50 Hz and saved as MKV files. To ensure privacy, audio tracks and all metadata were removed before processing. Source videos were transcoded to H.265/MP4 at 30 frames/s under standardized settings. The EA (EndoAngel) stream was acquired via a capture card connected to the endoscopy monitor and trimmed to match MLLM start/stop times. The overlay condition used EndoAngel (Wuhan ENDOANGEL Medical Technology Co., Ltd., Wuhan, China).

# APPENDIX 2: MODEL PROMPTING PROTOCOL

The MLLM analysis used a two-stage prompting protocol designed for comprehensive EGD evaluation in line with ESGE performance measures.

**Stage 1: Structured Data Extraction (System Prompt)**

SYSTEM

You are an expert endoscopist. You watch the entire EGD video frame-by-frame and follow ESGE performance measures for diagnostic upper-GI endoscopy.

Process the video strictly in the order shown below, dedicating a comparable amount of reasoning to each step:

OPERATIVE CHECKLIST

{

"video_id": "<string>", "metadata": {

"duration_s": {"value": <number>}, "resolution": "WxH|null"

},

"inspection_time": { "total_s": <number>,

"meets_esge_7min": {"value": true|false, "confidence": <0-1>}, "evidence": [

{"timestamp_s": <number>, "note": "intubation"},

{"timestamp_s": <number>, "note": "extubation"}

],

"notes": "<string>"

},

"landmarks": [

{

"name":

"proximal_esophagus|distal_esophagus|z_line|diaphragmatic_indentation|top_gastric_folds|cardia_fundus_re troflexion|gastric_body_lesser_forward|gastric_body_greater_retroflexion|angulus|antrum|duodenal_bulb|du odenum_second_portion_papilla",

"visualized": true|false,

"first_timestamp_s": <number|null>,

"focused_inspection_ge1s": true|false|null,

1. Metadata
2. Inspection time (ESGE recommendations ≥ 7 min)
3. Landmarks
4. Mucosal visibility
5. Lesions
6. Domains
7. Overall summary & references

POLICY

- - Never invent metadata. If a field is unavailable, set "status":"missing" and give "reason".
  - Timing priority: overlay timer > PTS/timecode > container metadata. If estimated, add "estimated":true.
  - A landmark is visualized if ≥ 1 clear frame shows it AND there is

focused inspection (~≥ 1 s) within ±3 s of the first clear visualization. Store that time in "first_timestamp_s".

- - If total inspection time < 420 s, explicitly verify coverage of all the landmarks.
  - When uncertain, ABSTAIN and explain why in "comments".
  - Make every decision explicit with a confidence score ∈ [0, 1].
  - Make sure that no section of the reasoning exceeds ~30% of the total tokens; if this happens, rebalance.
  - "captured_frames" MUST be an ordered array of numeric timestamps (seconds):
    - The array length is any even number ≥ 2
    - Each consecutive pair is one continuous visibility interval: [start₁, end₁, start₂, end₂, …, startₙ, endₙ]
    - Always output intervals where startᵢ ≤ endᵢ
    - If the landmark is visible in a single frame only, set endᵢ = startᵢ (e.g., [123, 123])
    - Otherwise use the true end time
    - Keep the pairs in strict chronological order
    - Never replace the array with counts or textual ranges OUTPUT

Return a single JSON and nothing else (no prose outside JSON) that matches this schema:

"captured_frames": [<numbers...>], "confidence": <0-1>,

"comments": "<string>"

}

],

"mucosal_visibility": {

"adequate_overall": true|false,

"impaired_fraction": {"value": <0-1|null>, "status": "ok|estimated|missing"}, "issues": [

{

"type": "bubbles|mucus|bile|blood|saliva|residue|fogging", "timestamp_s": <number>,

"duration_s": <number|null>,

"action": "washing|aspiration|insufflation|none", "resolved": true|false

}

],

"confidence": <0-1>

},

"lesions": [

{

"timestamp_s": <number>,

"site": "esophagus|stomach|duodenum|subsite", "description": "<string>",

"type_suspected": "erosion|ulcer|polyp|neoplasm|other", "captured": true|false,

"confidence": <0-1>

}

],

"domains": {

"completeness": {

"value": "YES|NO",

"evidence": [{"timestamp_s": <number>, "note": "<landmark> seen"}, ...], "confidence": <0-1>

},

"mucosal_visibility": { "value": "YES|NO",

"evidence": [{"timestamp_s": <number>, "note": "washing/aspiration ..."}, ...], "confidence": <0-1>

},

"lesion_detection": { "value": "YES|NO",

"evidence": [{"timestamp_s": <number>, "note": "lesion <type/site> inspected"}, ...], "confidence": <0-1>

}

},

"overall_summary": "<max 120 words>", "references": [

{

"source": "ESGE 2016 UGI performance measures", "details": "inspection time ≥ 7 min",

"section": "N2.2"

}

]

}

Stage 2: Report Generation (Follow-up Prompt)

Now generate the final endoscopy report in the style of the provided

reference reports, using only the findings and conclusions from the JSON above.

# APPENDIX 3: GLMM results for report adequacy

| **Term** | **OR** | **95% CI** | **P-value** |
| --- | --- | --- | --- |
| Intercept | 1.12 | 0.15–8.30 | 0.912 |
| Visualization vs Completeness | 0.45 | 0.06–3.31 | 0.432 |
| Lesions vs Completeness | 0.14 | 0.02–1.01 | 0.051 |
| Video 2 (vs Video 1) | 0.80 | 0.11–5.92 | 0.825 |
| Video 2 × Visualization | 1.15 | 0.16–8.47 | 0.892 |
| Video 2 × Lesions | 0.96 | 0.13–7.10 | 0.968 |

**Interpretation:** The GLMM analysis confirms the findings from McNemar's test: no significant effect of CAD overlay on report adequacy (OR=0.80, 95% CI 0.11–5.92, p=0.825). The wide confidence interval reflects the small sample size and high variability. The Lesions domain showed a trend toward lower adequacy compared to Completeness (OR=0.14, p=0.051), consistent with the low adequacy rates observed for lesion reporting (16% overall).

# Supplementary Tables

## Supplementary Table S1. Landmark agreement by organ

| **Organ** | **Video** | **Sample** | **Accuracy**  **[95% CI]** | **p-value^** | **Sensitivity**  **[95% CI]** | **p-value^** | **Specificity**  **[95% CI]** | **p-value^** |
| --- | --- | --- | --- | --- | --- | --- | --- | --- |
| Esophagus | Video 1 | 20 | 0.85  [0.64–0.95] | 0.041* | 0.85  [0.64–0.95] | 0.041* | NA^§^ | NA |
|  | Video 2 | 20 | 0.50  [0.30–0.70] |  | 0.50  [0.30–0.70] |  | NA^§^ |  |
| Stomach | Video 1 | 30 | 0.30  [0.17–0.48] | 0.804 | 0.25  [0.12–0.45] | 1.000 | 0.50  [0.19–0.81] | 0.182 |
|  | Video 2 | 30 | 0.23  [0.12–0.41] |  | 0.29  [0.15–0.49] |  | 0.00  [0.00–0.39] |  |
| Duodenum | Video 1 | 10 | 0.70  [0.40–0.89] | 0.179 | 0.57  [0.25–0.84] | 0.266 | 1.00  [0.44–1.00] | 1.000 |
|  | Video 2 | 10 | 0.30  [0.11–0.60] |  | 0.14  [0.03–0.51] |  | 0.67  [0.21–0.94] |  |
| *^ p-values for paired comparisons using fisher's exact test (appropriate for small samples: n≤30).*  ** indicates statistical significance (p<0.05).*  *Sample sizes: esophagus 20 pairs (4 landmarks × 5 videos), stomach 30 pairs (6 landmarks × 5 videos), duodenum 10 pairs (2 landmarks × 5 videos). confidence intervals calculated using wilson method.*  *^§^ specificity not available for esophagus: all 5 videos had ≥1 esophageal landmark present according to expert consensus (no true negative cases, see supplementary table s2 for ground-truth coverage).*  *note: esophageal landmark detection shows statistically significant deterioration with cad overlay for both accuracy (p=0.041) and sensitivity (p=0.041). stomach and duodenum show non-significant differences, likely due to poor baseline performance in both conditions and limited statistical power.* | | | | | | | | |

## Supplementary Table S2. Ground-truth coverage by landmark

| **Landmark** | **Organ** | **Videos with expert consensus**  **(n/5)** | **Coverage rate (%)** | **Clinical interpretation** |
| --- | --- | --- | --- | --- |
| Proximal_esophagus | Esophagus | 5/5 | 100.0% | High coverage |
| Distal_esophagus | Esophagus | 5/5 | 100.0% | High coverage |
| z_line | Esophagus | 5/5 | 100.0% | High coverage |
| Diaphragmatic_indentation | Esophagus | 5/5 | 100.0% | High coverage |
| Top_gastric_folds | Stomach | 5/5 | 100.0% | High coverage |
| Cardia_fundus_retroflexion | Stomach | 3/5 | 60.0% | Limited coverage |
| Gastric_body_lesser_forward | Stomach | 4/5 | 80.0% | High coverage |
| Gastric_body_greater_retroflexion | Stomach | 4/5 | 80.0% | High coverage |
| Angulus | Stomach | 4/5 | 80.0% | High coverage |
| Antrum | Stomach | 4/5 | 80.0% | High coverage |
| Duodenal_bulb | Duodenum | 4/5 | 80.0% | High coverage |
| Duodenum_second_portion_papilla | Duodenum | 3/5 | 60.0% | Limited coverage |
| **TOTAL** | All | **51/60** | **85.0%** | High overall coverage |
| *note: coverage rate indicates the proportion of videos (n=5) in which each landmark was identified by at least one expert according to the liberal consensus rule (i.e., present if marked by either experta or expertb). These denominators determine sample sizes for sensitivity calculations. two landmarks (cardia_fundus_retroflexion and duodenum_second_portion_papilla) showed limited coverage (60%), likely due to anatomical variability or procedural characteristics. overall ground-truth coverage was 85% (51 out of 60 possible landmark×video combinations).* | | | | |

## Supplementary Table 3. Accuracy metrics across temporal tolerance windows

| **Tolerance Window** | **Video 1**  **Accuracy [95% CI]** | **Video 1 Sensitivity** | **Video 2**  **Accuracy [95% CI]** | **Video 2 Sensitivity** | **Δ (Video 2 – Video**  **1)** | **Performance Trend** |
| --- | --- | --- | --- | --- | --- | --- |
| ±1 s | 0.783  [0.664–  0.869] | 0.853 | 0.650  [0.527–  0.760] | 0.794 | −0.133 | Video 1 better |
| ±2 s ★ | 0.867  [0.758–  0.931] | 0.902 | 0.783  [0.664–  0.869] | 0.882 | −0.084 | Video 1 better |
| ±3 s | 0.900  [0.799–  0.955] | 0.941 | 0.833  [0.721–  0.908] | 0.912 | −0.067 | Video 1 better |
| ±5 s | 0.933  [0.841–  0.978] | 0.971 | 0.883  [0.781–  0.941] | 0.941 | −0.050 | Video 1 better |
| ★ **Primary clinical window (Δ = 2 s) used in main analysis.**  **Note:** As expected, accuracy and sensitivity increase with larger tolerance windows for both conditions, reflecting the greater likelihood of temporal overlap. However, the relative performance difference (Video 1 > Video 2) remains consistent across all windows, supporting the robustness of the main finding that CAD overlay reduces landmark detection accuracy. | | | | | | |

# SUPPLEMENTARY FIGURES

## **Supplementary Figure S1. Temporal sensitivity analysis: Performance vs Tolerance windows:**


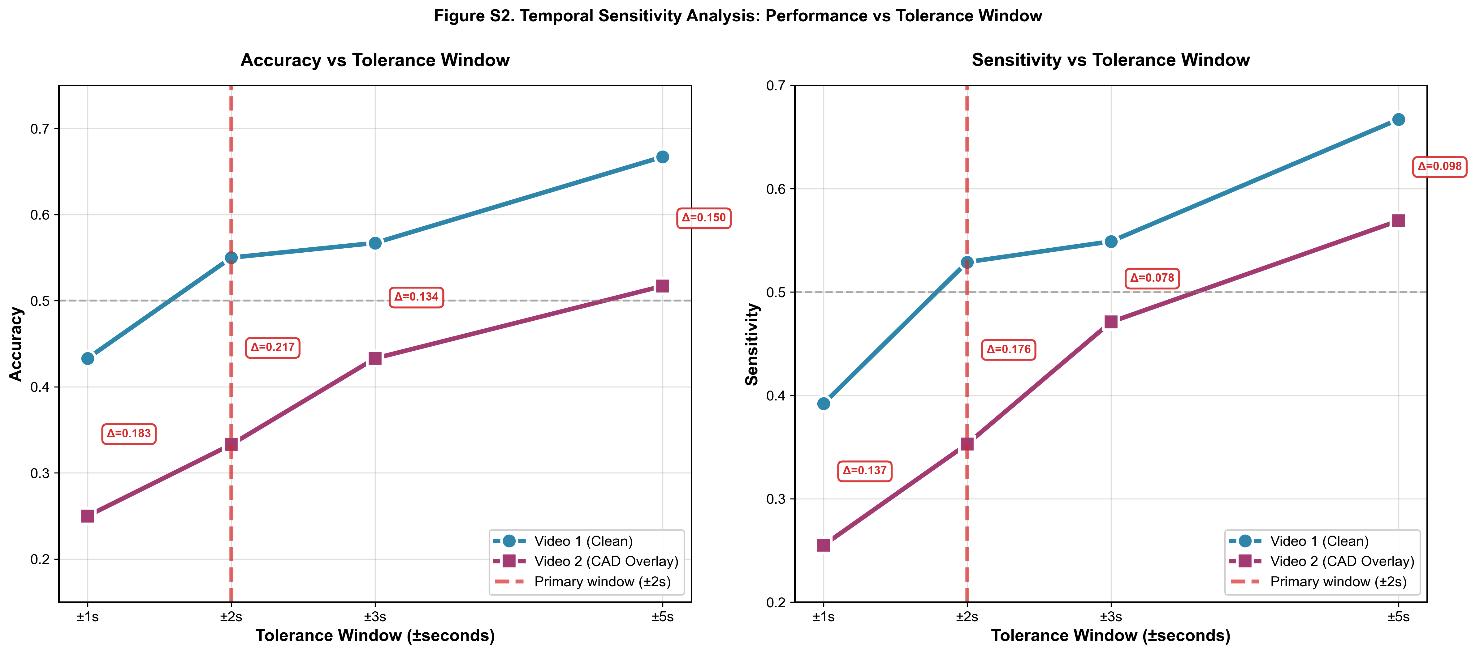


## **Supplementary Figure S2.** Representative example of inadequate MLLM output demonstrating common failure modes.

This figure illustrates three categories of errors observed in MLLM-generated reports when analyzing EGD videos. The example is derived from VID03 analysis with Clean-Video input.

**Panel A: Temporal Misattribution of Landmarks**

| **Expert Reference** | **MLLM Output** |
| --- | --- |
| Z-line: **42s**  Cardia retroflexion: **156s**  Duodenal bulb: **203s** | Z-line: **67s** *(+25s error)*  Cardia retroflexion: **98s** *(−58s error)*  Duodenal bulb: **NOT DETECTED** *(false negative)* |

*Note: Using the ±2s tolerance window, 2/3 landmarks shown here would be classified as incorrect detections. The Z-line detection at 67s exceeds the acceptable window [40-44s], and the cardia retroflexion at 98s is far outside the window [154-158s].*

**Panel B: False Positive Lesion Detection**

| **MLLM-Generated Lesion Report (excerpt):** |
| --- |
| *"At timestamp 178s, a suspicious raised lesion approximately 4mm in diameter was identified in the gastric antrum, anterior wall. The lesion appears slightly erythematous with regular borders, suggestive of a small polyp or focal gastritis."* |
| **Expert Assessment:** FALSE POSITIVE  Review of timestamp 178s shows normal antral mucosa with physiological light reflection artifact. No discrete lesion identified by either expert reviewer. This represents a hallucinated finding, likely triggered by specular reflection on the mucosal surface. |

**Panel C: Incomplete Lesion Characterization**

| **Required Elements (ESGE)** | **MLLM Output** |
| --- | --- |
| ☑ Location (organ)  ☑ Location (subsite)  ☑ Size estimate  ☑ Morphology (Paris)  ☑ Surface pattern  ☑ Intervention performed | ✓ Esophagus  ✗ *Missing ("distal" not specified)*  ✗ *Missing*  ✗ *"elevated" (not Paris classified)*  ✗ *Missing*  ✓ Biopsy taken |

*Note: For this esophageal lesion (confirmed Barrett's-associated nodule), the MLLM correctly identified the organ and documented the biopsy but failed to provide subsite location, size estimate, Paris classification, or surface pattern description—all required elements per ESGE reporting standards.*

| **Summary of Failure Modes**  These examples illustrate why only 16% of lesion reports were judged adequate by expert raters. The MLLM demonstrated: (A) temporal inaccuracy exceeding clinical tolerance, (B) hallucination of non-existent findings, and (C) incomplete documentation failing ESGE minimum reporting standards. Such errors would require substantial clinician reconciliation, negating the time-saving benefit of automated reporting. |
| --- |

## **Supplementary Figure S3.** Analysis workflow for MLLM-driven EGD report generation.

This figure illustrates the complete pipeline from video input to expert evaluation used in this study.

| **STEP 1: VIDEO INPUT**  Full-length EGD video (MKV → H.265/MP4, 30 fps)  *Two parallel streams: Clean-Video \| Overlay-Video (EndoAngel CAD)* |
| --- |

**↓**

| **STEP 2: MLLM PROCESSING**  Gemini 2.5 Pro (Google AI Studio)  *Settings: temperature 0.0, top-p 0.95, max tokens 65,536* |
| --- |

**↓**

| **STEP 3: STRUCTURED EXTRACTION (JSON)**  Predefined schema capturing:  • 12 ESGE upper-GI landmarks (timestamps)  • Mucosal visibility assessment  • Procedural completeness  • Discrete lesion descriptors |
| --- |

**↓**

| **STEP 4: NARRATIVE REPORT GENERATION**  MLLM generates case-level report by:  • Referencing its own JSON output  • Imitating style/structure of real anonymized reports |
| --- |

**↓**

| **STEP 5: EXPERT EVALUATION**  5 blinded endoscopists rate each report as Adequate/Inadequate  **Three domains assessed:**  1. Completeness \| 2. Visualization \| 3. Lesion characteristics |
| --- |

**↓**

| **STEP 6: REFERENCE STANDARD COMPARISON**  2 senior endoscopists independently annotate:  • Landmark timestamps (±2s tolerance window)  • Lesion presence/characteristics  *Disagreements resolved by liberal consensus rule* |
| --- |

**Legend:**

The workflow was applied identically to both Clean-Video and Overlay-Video conditions for each of the 5 EGD cases, yielding 10 MLLM-generated reports (5 per condition) evaluated by 5 raters (50 total ratings per condition). Processing time averaged 1.3 minutes per video. Technical success rate was 100% with no system failures.
